# Development and validation of a nomogram for predicting acute kidney injury after orthotopic liver transplantation

**DOI:** 10.1080/0886022X.2021.2009863

**Published:** 2021-12-04

**Authors:** Dandan Guo, Huifang Wang, Xiaoying Lai, Junying Li, Demin Xie, Li Zhen, Chunhui Jiang, Min Li, Xuemei Liu

**Affiliations:** Department of Nephrology, The Affiliated Hospital of Qingdao University, Qingdao, China

**Keywords:** Acute kidney injury, severe, liver transplantation, prediction nomogram, prognosis, survival, CKD

## Abstract

**Background:**

We aim to develop and validate a nomogram model for predicting severe acute kidney injury (AKI) after orthotopic liver transplantation (OLT).

**Methods:**

A total of 576 patients who received OLT in our center were enrolled. They were assigned to the development and validation cohort according to the time of inclusion. Univariable and multivariable logistic regression using the forward variable selection routine were applied to find risk factors for post-OLT severe AKI. Based on the results of multivariable analysis, a nomogram was developed and validated. Patients were followed up to assess the long-term mortality and development of chronic kidney disease (CKD).

**Results:**

Overall, 35.9% of patients were diagnosed with severe AKI. Multivariable logistic regression analysis revealed that recipients’ BMI (OR 1.10, 95% CI 1.04–1.17, *p* = 0.012), hypertension (OR 2.32, 95% CI 1.22–4.45, *p* = 0.010), preoperative serum creatine (sCr) (OR 0.96, 95% CI 0.95–0.97, *p* < 0.001), and intraoperative fresh frozen plasm (FFP) transfusion (OR for each 1000 ml increase 1.34, 95% CI 1.03–1.75, *p* = 0.031) were independent risk factors for post-OLT severe AKI. They were all incorporated into the nomogram. The area under the ROC curve (AUC) was 0.73 (*p* < 0.05) and 0.81 (*p* < 0.05) in the development and validation cohort. The calibration curve demonstrated the predicted probabilities of severe AKI agreed with the observed probabilities (*p* > 0.05). Kaplan-Meier survival analysis showed that patients in the high-risk group stratified by the nomogram suffered significantly poorer long-term survival than the low-risk group (HR 1.92, *p* < 0.01). The cumulative risk of CKD was higher in the severe AKI group than no severe AKI group after competitive risk analysis (HR 1.48, *p* < 0.05).

**Conclusions:**

With excellent predictive abilities, the nomogram may be a simple and reliable tool to identify patients at high risk for severe AKI and poor long-term prognosis after OLT.

## Introduction

Acute kidney injury (AKI) is a common and significant complication after orthotopic liver transplantation (OLT), which is the only available treatment for patients with end-stage liver disease [1]. Despite advances in organ preservation, surgical techniques, and improvements in immunosuppression programs, the incidence of post-OLT AKI remains high. Probably due to inconsistent definitions of AKI in different articles, the incidence of post-OLT AKI ranges between 5% and 95%, and 8%∼17% of these patients require renal replacement therapy (RRT) [2–4].

AKI has a significant influence on both short- and long-term prognosis in patients after OLT. Some researches indicated that the mortalities of in-hospital, 30-day, and 1-year after OLT were significantly higher in patients with AKI [5]. Post-OLT AKI may also progress to a requirement for postoperative RRT, CKD, even to end-stage renal disease (ESRD) [6]. Previous studies [6–10] have indicated that the long-term survival of graft and patients decreased significantly in recipients with severe AKI (KDIGO stage 2&3), compared to recipients with no or mild AKI. And severe AKI was correlated with a higher risk of CKD, heart failure, major atherosclerotic cardiovascular disease events, and all-cause death. Additionally, early initiation of CRRT was independently associated with survival benefits in recipients with severe AKI [11]. Therefore, early assessment of the risk for severe AKI is critical to improve patients’ prognoses.

The mechanism of severe AKI after OLT remains unclear. It is multifactorial involving preoperative, intraoperative, and postoperative factors. Known risk factors for AKI after OLT included recipients’ BMI [12], a history of diabetes or hypertension [13,14], the Model for End Stage Liver Disease (MELD) score [15], preoperative creatinine [3], and ischemia reperfusion injury (IRI) in the graft [16].

Although many risk factors for AKI and severe AKI have been identified, their cumulative effect remains unclear. Nomogram is a visual tool that integrates multiple factors, assisting clinicians to comprehensively predict the prognosis of patients and make clinical decisions. It has been applied to predict AKI in many other clinical settings, such as nephrectomy surgery [17], cardiac surgery [18], and intracranial aneurysm clipping surgery [19], but rarely in OLT recipients. Therefore, in order to make early diagnosis and develop interventions for patients at high risk for severe AKI after OLT, a nomogram was established in this study. By integrating these included clinical factors, the nomogram could provide clinicians with an individualized estimate of the probability of severe AKI after OLT.

## Methods and patients

### Study population

**Figure 1.**
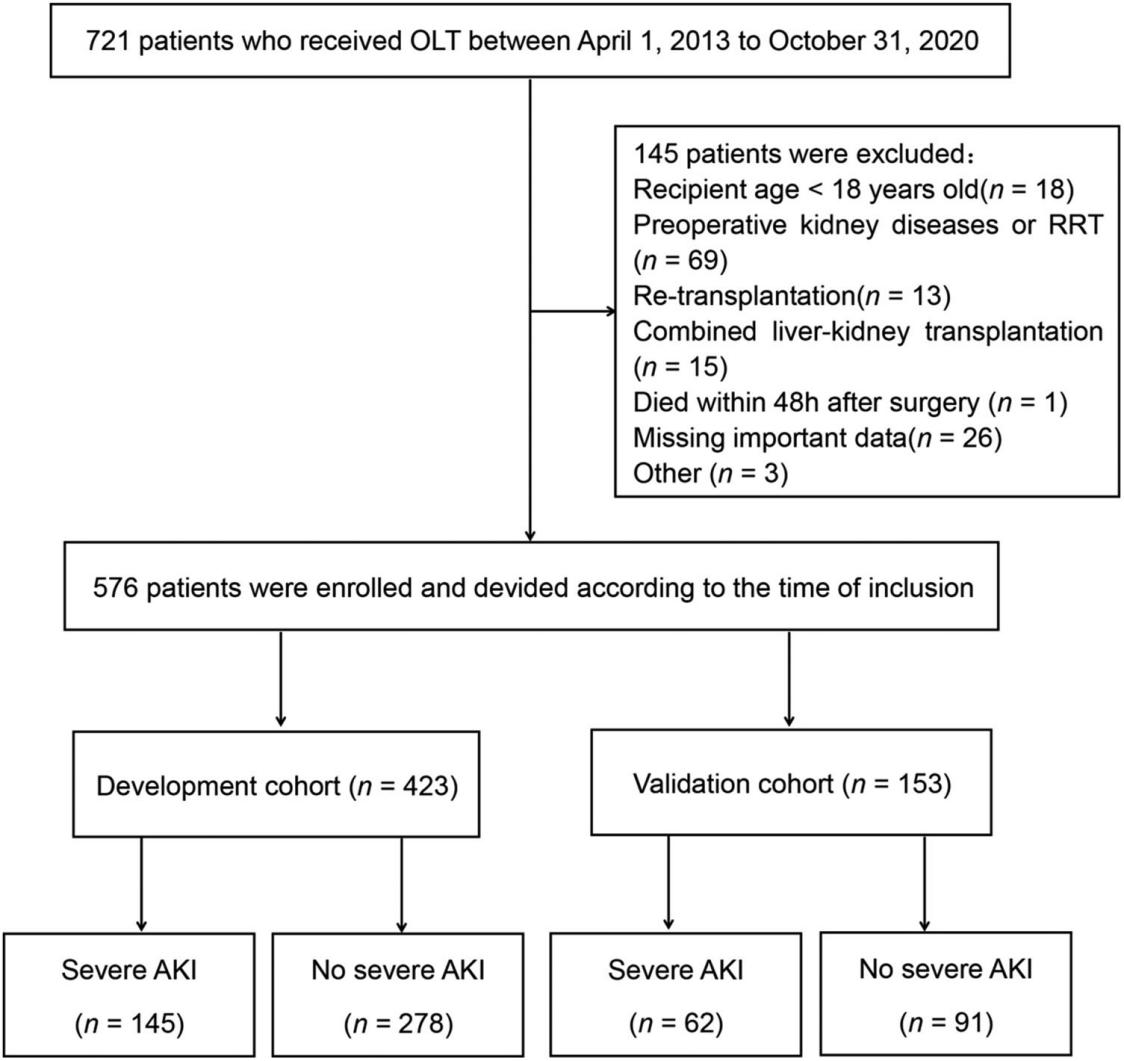
Flow diagram of patients enrollment.

Data from 721 patients who underwent OLT between 1 April 2013 to 31 October 2020 at the Affiliated Hospital of Qingdao University were retrospectively extracted from the electronic medical record system. Exclusion criteria included age <18 years old, diagnosed kidney diseases or RRT before OLT, fulminant hepatic failure, retransplantation, combined liver-kidney transplantation, died intraoperatively or within 48 h after surgery, and missing important data. Finally, a total of 576 patients were enrolled. The development cohort included patients who underwent OLT between 1 April 2013 and 30 April 2019, while the validation cohort was collected between 1 May 2019 and 31 October 2020 (Figure 1). The patients’ privacy will not be leaked because the related data were collected through the medical record numbers rather than their names. This study was approved by the Ethics Committee of the Affiliated Hospital of Qingdao University (the ethics approval number is QYFY WZLL 26283).

### Definition

Postoperative AKI was defined according to the KDIGO criteria [20]: increase in serum creatinine (sCr) ≥0.3 mg/dL (≥26.5 μmol/L) within 48 h or an increase in sCr to ≥1.5 times the baseline within the first 7 days after surgery. AKI was classified into 3 stages: stage 1, sCr increase to ≥26.5 μmol/L or increase to 1.5–1.9-fold from baseline; stage 2, increase to 2–2.9-fold; and AKI stage 3, increase to >3-fold or ≥4.0 mg/dL (≥353.6 μmol/L) or application of RRT. The maximum sCr level was recorded during the first 7 days after surgery and compared to the baseline level. The most recent preoperative laboratory results were used as the baseline. Stages 2&3 were called severe AKI. The formula for the Model for End-Stage Liver Disease (MELD) score is: 3.8 × log_e_(bilirubin [mg/dL]) + 11.2 × log_e_(INR) + 9.6 × log_e_(sCr [mg/dL]) + 6.4×(etiology: 0 if cholestatic or alcoholic, 1 otherwise) [21]. The estimated glomerular filtration rate (eGFR) was calculated according to the Modification of Diet in Renal Disease (MDRD) equation [22]. CKD was defined as eGFR <60 mL/min/1.73 m^2^ for 3 months, regardless of the presence or absence of structural kidney damage [23]. Hypertension was defined as the mean systolic BP ≥140 mm Hg, the mean diastolic BP ≥90 mm Hg, and/or current treatment with antihypertensive medication [24].

### Outcome

Previous studies [6–10] have indicated that mainly severe AKI (KDIGO stage 2&3) has an impact on the long-term survival and occurrence of CKD. Therefore, we chose severe AKI as an outcome event in this prediction model. The long-term prognosis of patients, focusing on mortality and the development of CKD, was also monitored. The follow-up was completed in March 2021 through outpatient or telephone consultations.

### Covariates

Preoperative recipient factors included sex, age, BMI, personal histories of smoking and alcoholism, comorbidities (e.g., hypertension or diabetes mellitus), etiologies for OLT (viral hepatitis, hepatocellular carcinoma, alcohol-related liver disease, cholestatic liver disease, and others), MELD score, Child-Pugh score, hepatic decompensation (e.g., encephalopathy and ascites), preoperative left ventricular ejection fraction (LVEF), and preoperative laboratory variables (routine blood test, coagulation markers, blood electrolytes , and blood biochemical examinations).

Intraoperative factors included surgical duration, anhepatic time, graft-recipient body weight ratio (GRWR), blood loss and average blood loss per kilogram of body weight, intraoperative medication (ulinastatin, furosemide, and insulin), amount of blood product transfused (RBC, fresh frozen plasma (FFP), cryoprecipitate, platelet concentrate, and salvage blood), fluid administration (crystalloid and colloidal solution).

Postoperative clinical outcome variables included the requirement for RRT, length of postoperative hospital stay, length of ICU stay, and mortality of in-hospital.

### Sample size

The effective sample size in prediction research (development and validation) was determined by the number of outcome events. It was defined to have at least 10 outcome events per variable (EPV) to ensure accuracy [25]. Based on the documented prevalence of severe AKI in the literature of 30.7–41% [7,26–28], especially the incidence of 35.6% in our center from the previous literature, we expected a 35% event rate for severe AKI in this study. In order to allow 10 or fewer predictors in the final multivariable logistic regression model, we estimated that 286 patients or more were required. Our sample size and the number of outcome events far exceed the EPV method and therefore was expected to provide reliable estimates.

### Statistical analyses

R version 4.0.3 (R Foundation for Statistical Computing) and STATA 15.0 (StataCorp Texas, TX) were used to analyze the data. The Kolmogorov-Smirnov test was performed to examine the normality of the data. Median and interquartile range (IQR) were used to describe continuous variables and comparisons were performed using the Mann–Whitney *U* test. Categorical variables were expressed in quantities and percentages and comparisons were made using the Chi-square test or Fisher’s exact test. The univariable logistic regression was used to evaluate the association between perioperative factors and severe AKI in the development cohort. Variables with *p* < 0.05 were included in the multivariable logistic regression. The final multivariable logistic regression model was seriously chosen by using forward stepwise regression with Akaike’s information criterion (AIC) as the stopping rule [29]. The AIC value for the final model was minimized with the fewest number of variables. Multicollinearity variables were not incorporated into the multivariable logistic regression analysis, which was estimated by the variance inflation factor (VIF) with a reference value of 10 [26]. For further analysis, a nomogram was developed based on the results of the multivariable logistic regression analysis. Then, we validated the predictive ability of this model by examining discrimination and calibration in the development cohort and validation cohort. The discrimination was quantified by the area under the receiver operating characteristic (ROC) curve (AUC). The calibration was evaluated by the calibration curve [30]. The long-term survival was estimated using Kaplan–Meier analysis, with comparisons between groups made using log-rank test. The CKD incidence and mortality before CKD were assessed by the cumulative incidence function methods and Fine - Gray models, with death handled as a competing event. The ‘rms’ package was used for the nomogram and calibration curve. The ‘survminer’ and ‘survival’ packages were used for Kaplan–Meier analysis. For all statistical analyses, *p*-values of < 0.05 were considered statistically significant.

## Results

### Basic characteristics

A total of 576 recipients were enrolled in the final study population, including 423 patients in the development cohort and 153 patients in the validation cohort. The basic characteristics of the recipients in the development and validation cohorts were shown in Table 1. According to the KDIGO criteria, the incidence of severe AKI was 34.3% and 40.5% in the development and validation cohort, respectively (*p* > 0.05).

**Table 1.** Perioperative characteristics of patients between development and validation cohort.

Characteristic	Development data (*n* = 423)	Validation data (*n* = 153)	*p*-Value
Demographic data			
Age (years)	53 (45–59)	52 (45–59)	0.44
Male (*N*,%)	347 (82.0%)	125 (81.7%)	0.90
BMI (Kg/m^2^)	24.09 (21.95–25.95)	24.24 (21.45–27.16)	0.36
MAP (mmHg)	91.33 (84.67–98.00)	91.33 (83.67–97.00)	0.68
Personal history			
Smoking (*N*,%)	191 (45.2%)	58 (37.9%)	0.13
Alcoholism (*N*,%)	191 (45.2%)	70 (45.8%)	0.92
Pathogenesis of liver disease			
HBV hepatitis (*N*,%)	316 (74.7%)	121 (79.1%)	0.32
HCV hepatitis (*N*,%)	9 (2.1%)	6 (3.9%)	0.24
Alcoholic liver cirrhosis (*N*,%)	47 (11.1%)	8 (5.2%)	0.037
Hepatocellular carcinoma (*N*,%)	200 (47.3%)	83 (54.2%)	0.16
Cholestatic disease (*N*,%)	30 (7.1%)	7 (4.6%)	0.34
Other (*N*,%)	43 (10.2%)	16 (10.5%)	0.88
Liver complications			
Encephalopathy (*N*,%)	55 (13.0%)	20 (13.1%)	1.00
Ascite*s* > 1L (*N*,%)	111 (26.2%)	37 (24.2%)	0.67
Baseline medical status			
Diabetes mellitus (*N*,%)	83 (19.6%)	24 (15.7%)	0.33
Hypertension (*N*,%)	53 (12.5%)	24 (15.7%)	0.33
Coronary heart disease (*N*,%)	13 (3.1%)	5 (3.3%)	1.00
Abdominal surgery history (*N*,%)	110 (26.0%)	32 (20.9%)	0.23
Preoperative LVEF (%)	62 (60, 65)	62 (60, 64)	0.37
Preoperative nonselective β receptor blockers (*N*,%)	22 (5.2%)	15 (9.8%)	0.055
Preoperative diuretics (*N*,%)	123 (29.1%)	47 (30.7%)	0.76
Preoperative scores			
MELD score	12.71 (9.73–17.95)	13.26 (9.68–18.28)	0.46
MEL*D* > 20 (*N*,%)	30 (19.6%)	84 (19.9%)	1.00
Child-Pugh score	9 (8–13)	9 (8–14)	0.58
Child class (*N*,%)			0.61
A	9 (2.1%)	5 (3.3%)	
B	204 (48.2%)	76 (49.7%)	
C	210 (49.6%)	72 (47.1%)	
Preoperative laboratory data			
White blood cell(× 10^9^/L)	3.58 (2.35–5.34)	3.38 (2.26–5.28)	0.50
Neutrophils(× 10^9^/L)	2.39 (1.5–3.82)	2.29 (1.42–3.77)	0.48
Lymphocyte(× 10^9^/L)	0.65 (0.4–1.06)	0.62 (0.41–0.98)	0.22
RBC (× 10^12^/L)	2.96 (2.45–3.57)	2.96 (2.49–3.59)	0.82
Hemoglobin (g/L)	91 (77–110)	91 (77–111)	0.91
Hematocrit (%)	26.9 (23–32)	27.1 (23.3–32.6)	0.76
Platelet (× 10^9^/L)	64 (39–111)	71 (43–108)	0.38
Total protein (g/L)	58.87 (54.21–63.3)	58.55 (54.6–62.7)	0.65
Albumin (g/L)	35.67 (31.87–39.02)	34.43 (32.32–37.49)	0.15
TBIL (μmol/L)	37.36 (21.6–95.07)	35.91 (21.44–82.12)	0.63
DBIL (μmol/L)	17.16 (9.55–49.89)	15.58 (9.58–43.6)	0.65
IBIL (μmol/L)	18.88 (11.62–45.07)	18.06 (11.51–40.93)	0.76
ALT (U/L)	27 (17–51)	26 (18–49)	0.71
AST (U/L)	38 (25–74)	37 (25–61)	0.31
LDH (U/L)	159 (133–193)	164 (134–203)	0.37
BUN (mmol/L)	4.58 (3.6–5.91)	4.5 (3.46–5.47)	0.25
sCr (μmol/L)	64 (51–77)	63 (50–77)	0.93
UA (μmol/L)	236 (177–306)	244 (187–305)	0.74
eGFR (mL/min/1.73 m^2^)	116.49 (93.66–151.55)	120.15 (90.35–147.30)	0.91
Prothrombin time (s)	16.3 (14.1–20.4)	16.3 (14.2–20.2)	0.90
INR	1.42 (1.2–1.79)	1.4 (1.2–1.82)	0.95
Fibrinogen (g/L)	1.57 (1.16–2.18)	1.56 (1.07–2.22)	0.51
APTT (s)	44.2 (36.7–56.1)	43.3 (37.3–55.1)	0.77
Thrombin time (s)	18.2 (16.5–20.2)	18.2 (16.9–20.5)	0.22
DD (μg/L)	790 (450–1970)	830 (420–1660)	0.76
K^+^ (mmol/L)	3.88 (3.58–4.21)	3.87 (3.59–4.13)	0.59
Na^+^ (mmol/L)	142 (139.5–144)	142 (140–144)	0.91
Cl^−^ (mmol/L)	107 (104–110)	107 (104–110)	0.72
Graft factor			
Estimated GRWR	2.00 (1.67–2.48)	2.04 (1.58–2.65)	0.58
Operation details			
Operation time (minutes)	490 (435–580)	490 (430–575)	0.97
Anhepatic time (minutes)	51 (45–58)	50 (45–59)	0.47
Estimated blood loss (mL)	1000 (800–2000)	1200 (800–2000)	0.56
Blood loss per body weight (mL/Kg)	17.14 (10.53–33.33)	17.65 (10.81–32.61)	0.71
Intraoperative fluid administration			
Crystalloid (mL)	4360 (3545–5345)	4250 (3560–5100)	0.48
Colloid (mL)	0 (0–0)	0 (0–0)	0.36
Total liquid inflow (mL)	4530 (3670–5585)	4375 (3630–5130)	0.27
Intraoperative transfusion			
RBC (units)	8 (4–12.5)	7.5 (4–11)	0.29
FFP (mL)	1000 (770–1570)	1000 (800–1570)	0.76
Cryoprecipitate (units)	0 (0–0)	0 (0–0)	0.81
PLT (units)	0 (0–0)	0 (0–0)	0.69
Salvage blood (mL)	0 (0–0)	0 (0–0)	0.88
Intraoperative drugs			
Ulinastatin (million units)	0 (0–30)	0 (0–30)	0.50
Furosemide (mg)	20 (5–70)	40 (17–80)	0.002
Insulin (IU)	0 (0–8)	0 (0–10)	0.37
Postoperative parameter			
Peak AST (U/L)	1017 (658–1936)	1177 (727–2157)	0.052
Postoperative RRT (*N*,%)	28 (6.6%)	17 (11.1%)	0.081
Postoperative hospital stay (day)	24 (21–31)	24 (20–30)	0.90
Length of ICU stay (day)	4 (3–6)	4 (3–6)	0.54
Died in hospital (*N*,%)	8 (5.2%)	12 (2.8%)	0.20
Secondary OLT (*N*,%)	2 (1.3%)	3 (0.7%)	0.61

Continuous variables are displayed as median and interquartile ranges.

BMI: Body mass index; MAP: Mean arterial pressure; LVEF: Left ventricular ejection fraction; MELD: Model for End-Stage Liver Disease; RBC: Red blood cell; TBIL: Serum total bilirubin; DBIL: Serum direct bilirubin; IBIL: Serum indirect bilirubin; ALT: Alanine aminotransferase; AST: Aspartate aminotransferase; LDH: Lactate dehydrogenase; BUN: Blood urea nitrogen; sCr: Serum creatinine; UA: Uric acid; eGFR: Estimated glomerular filtration rate; INR: International normalized ratio of prothrombin time; APTT: Activated partial thromboplastin time; DD: D dimer; GRWR: Graft-recipient body weight ratio; FFP: Fresh frozen plasma; Peak AST: AST peak value within first 24 h after OLT.

The median age of all recipients was 52 (45–59) years, with 81.9% were male. The most common etiology for OLT was hepatocellular carcinoma combined with HBV (45.4%) followed by HBV-related hepatic cirrhosis (26.9%). Overall, 71.4% of patients were subsequently diagnosed with AKI after OLT, and the incidence of stage 1, stage 2, and stage 3 AKI was 35.8%, 20.3%, 15.6%, respectively. 7.8% of patients required postoperative RRT. 44.3% of AKI occurred at the first postoperative day (POD), 25.8%, 13.2%, 6.8%, 3.5%, 4.0% and 2.4% of AKI occurred at POD 2 to POD 7, respectively.

### Logistic regression analysis for predictors of severe AKI after OLT

Univariable logistic analysis was performed in the development cohort to select risk factors for post-OLT severe AKI. The variables with *p* < 0.05 and several factors with clinical significance in previous researches were all included in the multivariable logistic regression, such as operation time and RBC transfusion, etc [7,31]. Afterward, the final regression model was obtained according to the minimum value of AIC with the fewest number of variables. Finally, recipients’ BMI (OR 1.10, 95% CI 1.04*–*1.17, *p* = 0.012), hypertension (OR 2.32, 95% CI 1.22–4.45, *p* = 0.010), preoperative sCr (OR 0.96, 95% CI 0.95–0.97, *p* < 0.001), and intraoperative FFP transfusion (OR for each 1000 ml increase 1.34, 95% CI 1.03–1.75, *p* = 0.031) were independent risk factors for post-OLT severe AKI (Table 2).

**Table 2.** Univariable and multivariable logistic regression analysis for predictors of severe AKI.

Variables	Univariable logistic regression			Multivariable logistic regression		
	*β*	*OR*(95% *CI*)	*p*	*β*	*OR*(95% *CI*)	*p*
Age	0.0121	1.01(0.99–1.03)	0.265			
BMI (Kg/m^2^)	0.0857	1.09(1.03–1.15)	0.003	0.0965	1.10(1.04–1.17)	0.002
MAP (mmHg)	0.0217	1.02(1.00–1.04)	0.021			
Hypertension	0.6206	1.86(1.04–3.32)	0.036	0.8451	2.32(1.22–4.45)	0.010
MELD score	0.0326	1.03(1.00–1.06)	0.027			
Operate time (minutes)	0.0006	1.00(0.99–1.00)	0.342			
GRWR	−0.0749	0.93(0.88–0.98)	0.010			
Blood loss (mL)	0.0001	1.00(0.99–1.00)	0.080			
RBC transfusion (units)	0.0109	1.01(0.98–1.04)	0.432			
FFP transfusion (per 1000 mL)	0.3004	1.35 (1.05–1.73)	0.017	0.2943	1.34(1.03–1.75)	0.031
Furosemide (per 100 mg)	0.4607	1.59(1.17–2.15)	0.003			
TBIL (μmol/L)	0.0014	1.00(1.00–1.00)	0.046			
DBIL (μmol/L)	0.0017	1.00(0.99–1.00)	0.127			
IBIL (μmol/L)	0.0045	1.00(1.00–1.01)	0.012			
BUN (mmol/L)	−0.0991	0.91(0.83–0.98)	0.024			
sCr (μmol/L)	−0.0348	0.97(0.95–0.98)	<0.001	–0.0415	0.96(0.95–0.97)	<0.001
UA (μmol/L)	−0.0032	0.99(0.99–1.00)	0.002			
eGFR (ml/min/1.73 m^2^)	0.0127	1.01(1.01–1.02)	<0.001			
INR	0.0922	1.09(0.90–1.32)	0.344			
Fibrinogen (g/L)	−0.1940	0.82(0.66–1.03)	0.087			
APTT(s)	0.0105	1.01(1.00–1.02)	0.014			
Peak AST (per 1000 U/L)	0.2453	1.28(1.12–1.46)	<0.001			

OR: Odds ratio; CI: Confidence interval; BMI: Body mass index; MAP: Mean arterial pressure; MELD: Model for End Stage Liver Disease; GRWR: Graft-recipient body weight ratio; RBC: Red blood cell; FFP: Fresh frozen plasma; TBIL: Serum total bilirubin; DBIL: Serum direct bilirubin; IBIL: Serum indirect bilirubin; BUN: Blood urea nitrogen; sCr: Serum creatinine; UA: Uric acid; eGFR: Estimated glomerular filtration rate; APTT: Activated partial thromboplastin time; peak AST: AST peak value within first 24 h after OLT.

### Development and validation of the nomogram model

**Figure 2.**
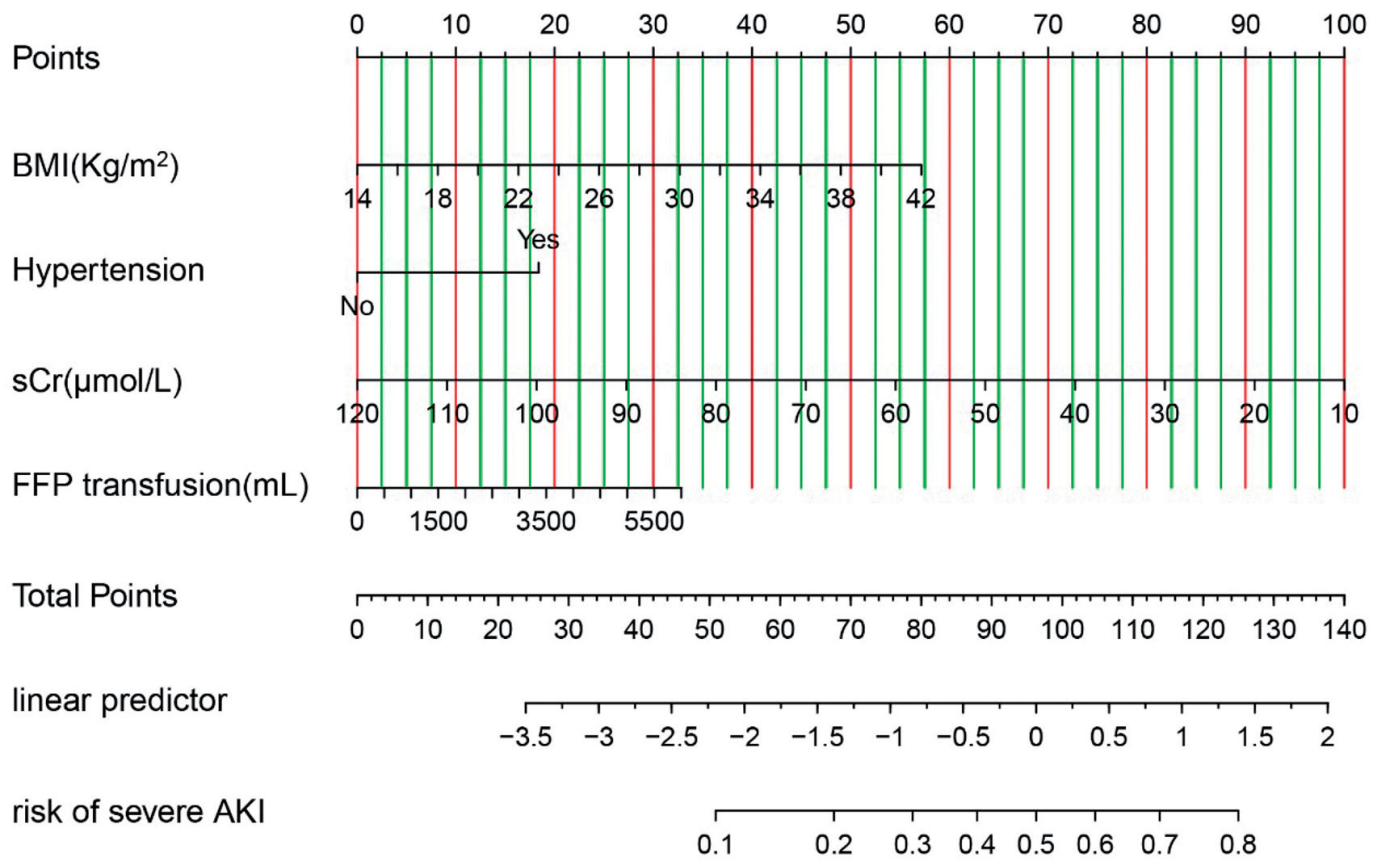
Nomogram for the prediction of severe AKI after OLT. Draw a vertical line toward the ‘Points’ axis to determine the points of each variable, add up the points and position it on the ‘Total Points’ axis. Draw a vertical line toward the ‘Risk of severe AKI’ axis to find the possibility of severe AKI after OLT. AKI: Acute kidney injury; BMI: Body mass index; sCr: Preoperative serum creatinine; FFP: Fresh frozen plasma.

**Figure 3.**
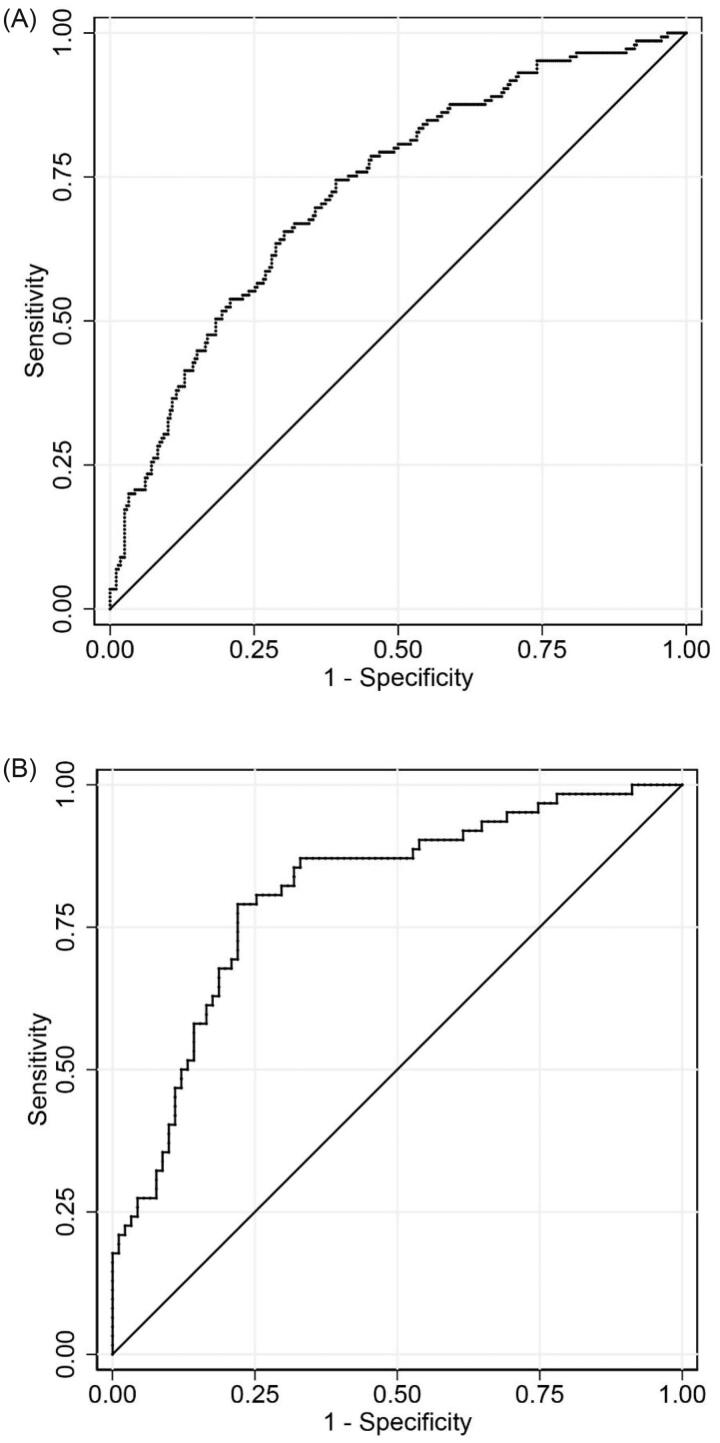
The ROC curve of the model forecasting the occurrence of severe AKI after OLT. The area under the ROC curve (AUC) were 0.73 (95% CI: 0.68–0.78, *p* < 0.05) in the development cohort (A) and 0.81 (95% CI: 0.74–0.88, *p* < 0.05) in the validation cohort (B).

**Figure 4.**
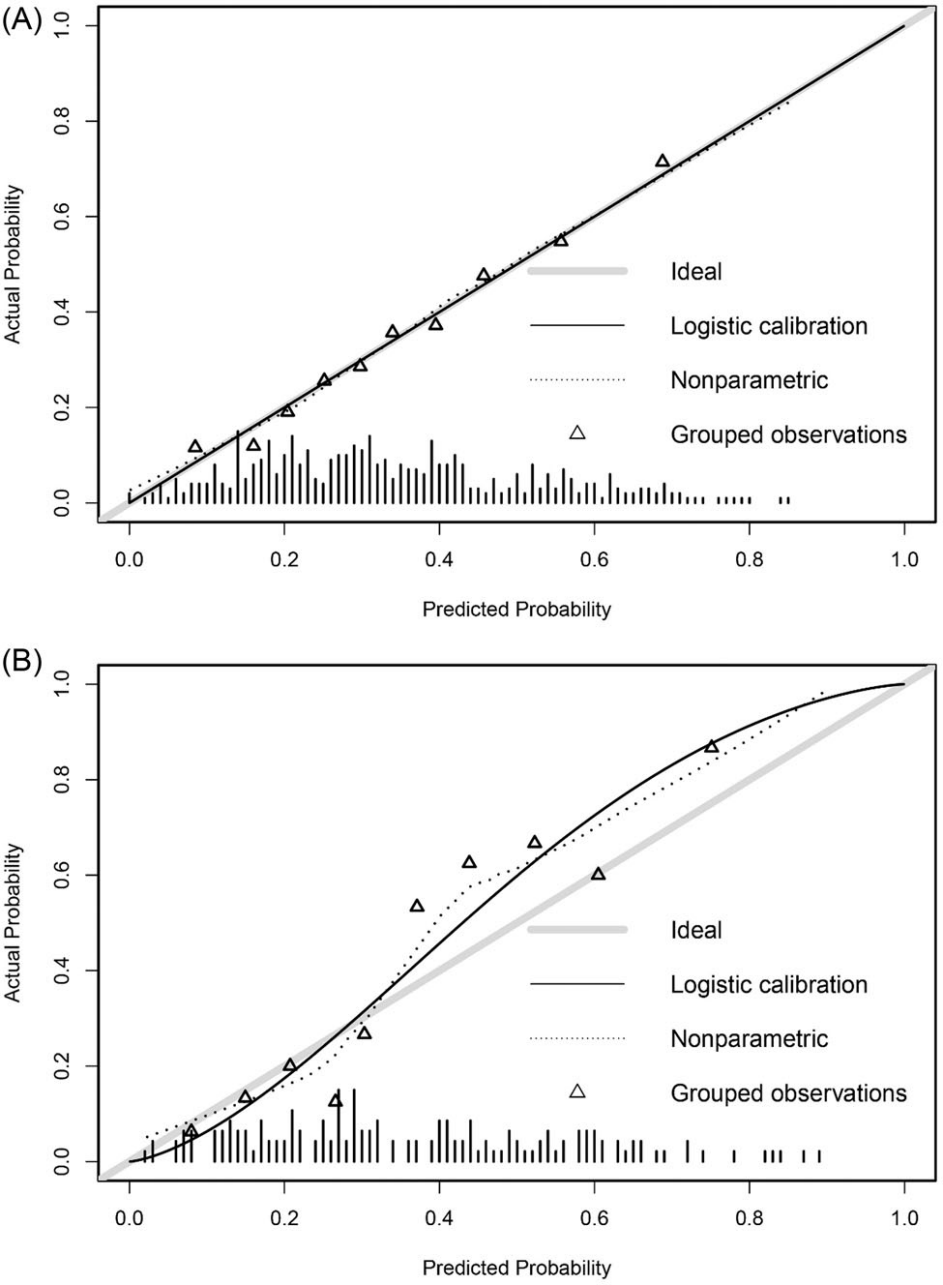
Calibration plot of predicted probability of severe AKI predicted by the nomogram model vs. observed probability in the development cohort (A) (*p* > 0.05) and validation cohort (B) (*p* > 0.05).

A nomogram for predicting the possibility of severe AKI after OLT was formulated using the results of the multivariable logistic regression (Figure 2). Points were assigned to the four identified predictors based on their regression coefficients. Add up the points of each factor decided by individuals to calculate the estimated possibility of severe AKI after OLT. Then, the nomogram was validated in the development cohort and validation cohort. The discrimination was quantified with area under the ROC curve (AUC), which was 0.73 (95% CI: 0.68–0.78, *p* < 0.05) in the development cohort and 0.81 (95% CI: 0.74–0.88, *p* < 0.05) in the validation cohort (Figure 3). The optimal cutoff value of the nomogram model to identify patients at high-risk for severe AKI was 0.36 according to the Youden index, which was determined based on the optimization of sensitivity and specificity through ROC analysis. The sensitivity and specificity of the model were 0.66 and 0.70 in the development cohort, and 0.79 and 0.78 in the validation cohort. The calibration curve of the nomogram was presented in Figure 4, which demonstrated that the severe AKI probabilities predicted by the nomogram agreed with the observed probabilities (*p* > 0.05). These results indicated that the nomogram model could accurately predict the risk of severe AKI after OLT.

### Short-term prognosis of patients with and without severe AKI

The in-hospital mortality of patients with severe AKI was approximately 4.25 times that of patients without severe AKI (*p* = 0.003). Postoperative RRT was required in 14.0% of patients with severe AKI and 4.3% of patients without severe AKI (*p* < 0.001). When compared with patients without severe AKI, the ICU stay was significantly longer in those with severe AKI (*p* = 0.002).

### Long-term prognosis of patients with and without severe AKI

**Figure 5.**
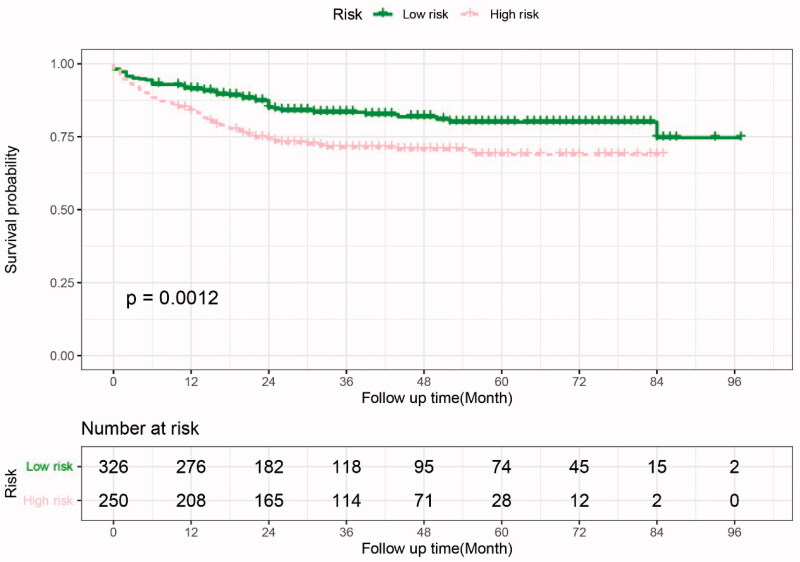
Patient survival after liver transplantation. Patients were divided into low-risk and high-risk groups at the probability value of 0.36 predicted by the nomogram. The long-term survival outcome was significantly poorer in the high-risk group than in the low-risk group (*p* < 0.05).

**Figure 6.**
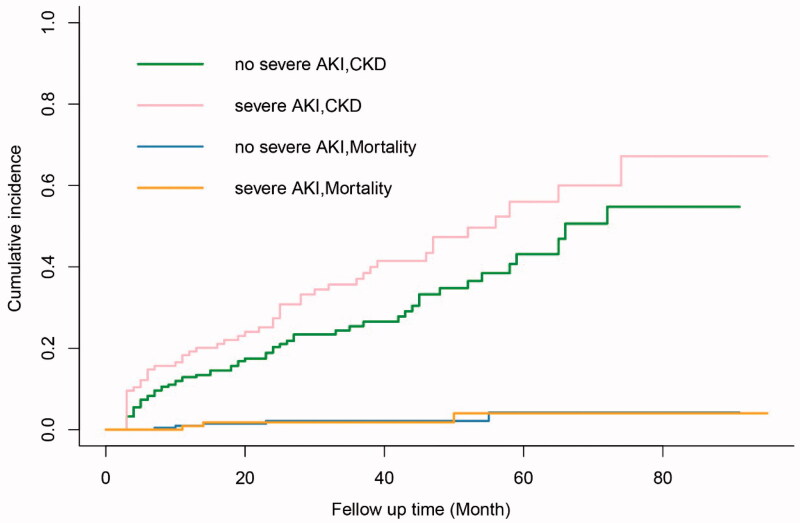
Cumulative incidence of CKD and competitive mortality in patients with and without severe AKI (*p* < 0.05).

Based on the probability of severe AKI predicted by the nomogram model, we further divided the patients into low- and high-risk groups with a cutoff value of 0.36. After a median follow-up period of 29.5 months (interquartile range: 16–52 months), the mortality was 28.4% and 15.6% in the high- and low-risk groups, respectively. Kaplan-Meier survival analysis with log-rank test showed that patients in the high-risk group had a significantly poorer long-term survival outcome than those in the low-risk group (HR 1.92, *p* < 0.01, Figure 5). Competing risk analysis showed that the incidence of CKD was significantly higher in the severe AKI group than the no severe AKI group after controlling for competitive mortality before CKD (HR 1.48, *p* = 0.039) (Figure 6). However, there was no statistical difference in the cumulative incidence of CKD between the high- and low-risk groups (*p* = 0.491).

## Discussion

In this study, we have developed and validated a nomogram model for predicting severe AKI after OLT for the first time. With excellent discrimination and calibration, it can be applied immediately at the end of surgery, assisting clinicians to identify patients at high risk for severe AKI and make preventive and therapeutic measures in advance. The risk factors enrolled in our model could also be collected from other centers easily, demonstrating the generalizability of this nomogram. Besides, we further divided the patients into low- and high-risk groups based on the probability of severe AKI predicted by the nomogram model. Patients in the high-risk group had a significantly poorer long-term survival outcome than those in the low-risk group. Therefore, this nomogram can also be used to predict long-term survival in advance and assist clinicians to pay more attention to these recipients during follow-up.

Postoperative AKI is a common complication after OLT. According to a relatively lower diagnostic threshold of KDIGO criteria, 71.4%, 35.9%, and 7.8% of these 576 recipients developed AKI, severe AKI, and required postoperative RRT after OLT, respectively. These results were consistent with previous studies that used the same KDIGO criteria, interpreting the generalizability of this nomogram [7,28,32]. The in-hospital mortality of patients with severe AKI was 4.25 times that of patients without severe AKI. And ICU stay was significantly longer in the severe AKI group. As for long-term prognosis, we further divided the patients into high- and low-risk groups based on the nomogram model. Compared with the low-risk group, the survival rate of patients in the high-risk group was significantly lower. However, the prevalence of CKD was not statistically different between the high- and low-risk groups. This might be related to the low incidence of CKD and the short follow-up period in this study. And the significant difference in the mortality of patients between the two groups might affect these results. It was not unexpected that the cumulative risk of CKD was significantly higher in the severe AKI group, indicating that the severe kidney injury may be persistent, long-term, and subsequently contribute to the progression to CKD.

As mentioned above, severe AKI after OLT is an important risk factor for the short and long-term prognosis. However, neither pharmacological nor non-pharmacological interventions have shown any significant benefits in preventing AKI after OLT. It is necessary to identify patients who are at high risk for severe AKI so that earlier protective strategy can be implemented.

Based on the logistic regression analysis, a risk prediction nomogram was developed using routine perioperative parameters. The recipients’ BMI, hypertension, preoperative sCr, and intraoperative FFP transfusion were identified as the independent risk factors for severe AKI. The strongest predictor in our model was hypertension. Hypertension has been a known risk factor for postoperative AKI. It was proposed that patients developing AKI were more likely to have arterial hypertension preoperatively, of whom the variation of blood pressure may be more significant [13]. And the greater difference between baseline and intraoperative blood pressure might exacerbate intraoperative hemodynamic disturbances, which could promote the occurrence of AKI. In addition, as a recognized feature of CKD, preoperative hypertension may be related to more advanced preexisting renal damage, which in itself was a potential risk factor for AKI after OLT [33].

The second predictor was recipients’ BMI, which was a known factor that increased the risk of AKI after OLT [7]. There were several explanations for the correlation. Firstly, obesity could lead to chronic mild inflammation associated with an increase in adipokines, which was reported to play an essential role in the pathogenesis of acute ischemia/reperfusion injury in the kidney. Adipokines could activate NF-κB and increase the expression of some proinflammatory molecules, such as TNF-α, IL-6, MIP-2, and MCP-1, which would lead to the infiltration of neutrophils, T cells, and macrophages into the injured kidneys [34]. Secondly, patients with elevated BMI had more severe oxidative stress and endothelial dysfunction, which were more predictive of postoperative AKI than inflammatory markers [35]. In addition, as a component of metabolic syndrome and an important risk factor for hypertension, diabetes, CKD, and cardiovascular disease, obesity may provide insufficient reserve during surgery to cope with stress-induced renal hypoperfusion [36]. In other researches, the impact of BMI on severe AKI remains controversial because obese or emaciated are both harmful to postoperative recovery. Consistent with previous research conducted in this center [32], emaciation has no effect on severe AKI, which might be related to the characteristics of our study population. There were only 5.0% of patients assigned to the underweight group (BMI < 18.5 kg/m^2^). And the mean value of BMI in this group was 17.3 kg/m^2^, approaching the lower limit of normal weight (18.5 ≤ BMI <25 kg/m^2^). Therefore, the influence of emaciation on severe AKI needs to be further analyzed by expanding the sample size.

Preoperative sCr level was significantly associated with the development of severe AKI after OLT. As reported previously, sCr was a key component of the MELD score, which also reflected the significance of renal function as a predictor for short-term survival in patients with liver disease. Nevertheless, there were still conflicting evidence regarding the impact of renal insufficiency before OLT on the development of AKI after OLT [37]. In this study, the preoperative sCr level was inversely associated with the occurrence of postoperative AKI. This result was consistent with previous studies [38,39]. One possible explanation for this phenomenon may be that in an era of increasing demand for organ transplants, high-quality grafts with a lower donor risk index were matched to the higher-risk recipients [40]. The discrepancy between the number of OLT candidates and the availability of liver grafts has led to the use of increasingly higher risk grafts that were not utilized in the past to reduce waiting list mortality [41]. More importantly, poor protein intake, reduced muscle mass, severe hyperbilirubinemia, volume expansion, and reduced liver synthesis of sCr are all contributed to the lower sCr level in liver disease [42], which possibly indicated that lower sCr may reflect more severe condition to some extent. Further researches are needed to confirm the specific mechanism.

The requirement for FFP during OLT was independently associated with post-OLT AKI, which was consistent with previous studies [28,43]. The reason for this result may be that recipients often experience massive blood loss due to portal hypertension and coagulation dysfunction during OLT. Additionally, coagulation dysfunction and fibrinolysis reflect severe hepatic IRI and early impaired graft function.

Although gender, diabetes mellitus, Child-Pugh score, and blood loss may be risk factors for AKI after OLT in several previous reports, they were not associated with the risk of severe AKI in this study [44,45]. The main reason may be related to the different endpoints between this and previous studies. However, as an important element of the MELD score and Child-Pugh score, the preoperative sCr level was incorporated into the final model. And the intraoperative FFP transfusion which reflected the abnormal coagulation mechanism was related to the blood loss. These results are consistent with several previous studies, suggesting that the MELD score, Child-Pugh score, and blood loss by themselves may not contribute to the development of severe AKI independently. As an indicator of renal and liver function before surgery, the sCr level may be more representative in predicting postoperative severe AKI. Furthermore, this could also be the result of the homogenization of our population with relatively low MELD scores, low prevalence of diabetes mellitus, and the majority of recipients were male in this study.

Finally, a nomogram was constructed based on the logistic regression analysis. It provided clinicians with a visual tool to understand the impact of predictors on the outcomes of postoperative severe AKI. We can accurately calculate the probabilities of severe AKI for individuals, making the results more personalized. The high AUC indicated the nomogram model has a strong ability to distinguish between patients with and without severe AKI. The excellent calibration curve showed the accurate prediction ability of this model. In addition, this model could identify patients with poor long-term outcomes, assisting clinicians to formulate interventions in advance to improve the prognosis of patients. Furthermore, the long-term value of this nomogram is that it can guide OLT in the future. For patients preparing for liver transplantation, their BMI and blood pressure should be intervened to achieve the target levels as soon as possible to reduce the risk of severe AKI after OLT.

There were several prediction models for AKI after OLT. A risk score for AKI after OLT in a Korean center [46] and another one from a Chinese multicentre [3] both used the old RIFLE-criteria for classifying AKI, which have been replaced by the latest KDIGO standards over the past few years. A nomogram model for predicting AKI after OLT has also been developed in the past but the sample size was too small [13]. Another nomogram was constructed based on the intraoperative hepatic blood inflow (HBI) to identify posttransplant AKI from a Chinese center. Nevertheless, the HBI could not be easily acquired in other centers, which limited the general use of this model [47]. A new model score for recipients of deceased donor transplantation from two European centers has been developed to identify patients at risk for severe AKI after OLT. This model score had good discriminative ability but it has not been used in Chinese patients [28].

Compared with these models, this model had the following advantages. In this study, the sample size was large enough and AKI was determined according to the KDIGO definition, which was reported to provide better prognostic capacity than the RIFLE and AKIN definitions [48]. Based on the perioperative variables that can be easily acquired, we constructed a nomogram model and validated it in the development and validation cohort, respectively. It was proved that this nomogram model has a strong discriminative ability and accurate predictive ability. Moreover, this prediction model could be used immediately at the end of operation, assisting clinicians to recognize recipients at high risk for severe AKI and poor long-term prognosis in advance. By considering early RRT and adjusting immunosuppression for these patients, the prognosis of the kidney may be improved. Furthermore, this new model was simple and easy to be popularized.

This study had several limitations. Firstly, this study was a retrospective single-center design, which should be evaluated in a prospective cohort in our center or in other centers to demonstrate its applicability. Secondly, the recipient, graft, and perioperative factors contributing to AKI have been analyzed, but the information of the donor was not included due to the privacy protection policy for the donor and recipient in our center. Thirdly, due to the difficulty in data collection, the relationship between other factors and postoperative AKI were did not analyzed, such as intraoperative hemodynamic parameter, dosage or duration of drug treatment, intraoperative and postoperative urine output. Moreover, postoperative transfusions, infection, and usage of contrast agents which would affect the incidence of AKI should also be included in the future. Besides, to accurately clarify the impact of AKI on the long-term prognosis after OLT, it is necessary to conduct a longer follow-up study.

## Conclusion

With excellent discrimination and calibration, a new nomogram model for predicting severe AKI after OLT was successfully developed and validated. It can be applied immediately at the end of the surgery, assisting clinicians to identify patients at high risk for severe AKI and poor long-term outcomes. By considering early RRT and adjusting the immunosuppression strategy in advance, the kidney prognosis may be improved.
